# The Hospital Nursing Supplement

**Published:** 1894-11-03

**Authors:** 


### "The Hospital" Nursing Mirror.

Being the Extra Nursing Supplement of "The Hospital" Newspaper.

[Contributions for this Supplement should be addressed to the Editor, The Hospital, 428, Strand, London, W.C., and should have the word "Nursing" plainly written in left-hand top corner of the envelope.]

### News from the Nursing World.

### OUR CHRISTMAS COMPETITIONS.

We trust that a great many of our readers are mindful of our annual Needlework Competition, and that they will induce their friends to take a practical interest in our plan of giving warm garments to the sick poor, whose Christmas Day is spent in hospital. Even the busiest people can surely spare some odd half-hours during the next two months to bring gladness and comfort to those who have but little of either. As we suggested the other day, readers with means, unable or unwilling to sew, might advantageously provide materials to be made up by those who have time and ability for work, and yet are unable to indulge in purchases. The prizes offered are: 20s. for the most serviceable dressing gown; 10s. for the best flannel shirt; 7s. 6d. for the best flannel petticoat; 7s. 6d. for the best over-petticoat; 7s. 6d. for the best bed jacket; 5s. for the best knitted pair of men's socks; and 2s. 6d. for the second best pair. We hope those who do not care to compete will contribute useful garments of all descriptions, and send them to 428, Strand, by December 18th.

### THE TRAINED NURSES' CLUB.

A pleasant gathering of matrons, nurses, midwives, and other guests took place at 12, Buckingham Street, on October 26th, when the opening of the new club rooms was made the occasion of an "At Home" given by Miss Wilson, President of the Institute and Hon. Secretary of the Workhouse Infirmary Nursing Association. The entertainment was a most successful one, an excellent programme, arranged by Mrs. Nichol, the popular Hon. Secretary, included a capital selection of music and various recitations. A dialogue, entitled, "A Funny Household," was given with great spirit, and the guests separated regretfully, feeling that the evening had passed away far too quickly. The new rooms form a valuable addition to the resources of the club, which is a most convenient and unpretentious one. For years it has gone on quietly, doing an amount of work of which few people who are not members have any conception.

### CHOLERA AND COMMON SENSE.

Assuming that existing nursing organisations provide for emergencies, in which, of course, epidemics must be included, The Hospital has always advocated the maintenance of uniform efficiency in the ranks of nurses rather than intermittent and sensational movements. The stray cases of cholera, cropping up annually where least expected, show that local officers of health and medical men are the best protectors both of public safety and patients' welfare. The Executive Committee of the Royal British Nurses' Association have presumably arrived by a different path at the same conclusion as ourselves, for they announce that "No call has been made upon the Roll of Cholera Nurses since it was instituted in the autumn of the year 1892," and "point out that such a result is due to that admirable efficiency of the sanitary service of the country."

### ADDENBROOKE'S HOSPITAL, CAMBRIDGE.

We congratulate all connected with this institution upon the decision arrived at by the Court of Governors on the 29th ult. On the motion of the Rev. S. G. Wood, seconded by Dr. Macalister, and supported by Dr. Latham and Dr. Hill, the governors unanimously resolved "to instruct the committee to further consider the matter of providing increased accommodation for the probationers, and in case the committee reports in favour of the employment of ward-maids to consider the question of accommodation for the servants of the hospital, to obtain detailed plans, quantities and estimates, and to report to the quarterly court at Christmas." The senior physician, the senior surgeon, the Rev. H. Hall, and Dr. Besant were added to the committee. It may now be hoped that Addenbrooke's Hospital, Cambridge, will speedily be brought into line with the best administered institutions so far as its nursing is concerned. How it has been possible for the present bad system in regard to probationer nurses to continue so long we are at a loss to imagine, but now that with the unanimous and hearty co-operation of the medical staff and the cordial support of the governors it has been decided to abolish it we may confidently expect the early inauguration of changes so complete as to make Addenbrooke's Hospital one of the most popular schools for training nurses in the United Kingdom.

### FLEETWOOD COTTAGE HOSPITAL.

The bazaar in aid of the building fund for the new cottage hospital at Fleetwood passed off most successfully, and the ladies who organised it may be congratulated on the handsome contribution which they were enabled to hand over. The hospital, which was opened last week by Mr. W. B. Ferguson in the presence of a large gathering of friends, will soon be ready for occupation.

### WOMEN STUDENTS.

Of the fifteen women students who entered for the first examination in medicine at the Glasgow University, twelve were successful in one or more subjects. Only one candidate was under the old regulations, and she too was successful. In the second examination three women passed, and in the third the same number. The degree in medicine and surgery will be conferred by the University this month on two students of Queen Margaret College. The friends of this college have succeeded in establishing a residence for women, under the title of Queen Margaret Hall, where eighteen students already in residence appear appreciative of the comfortable arrangements made for them.

### NURSING AT JEDBURGH.

The annual meeting of the Jedburgh Nursing Association was recently held in the Port Hall and was very well attended. Much appreciation was shown of the kind and willing work of Nurse Rae who attended 100 cases during the year, paying 2,500 visits. The guild for supplying medical comforts to the sick poor has proved a most valuable adjunct to the nurse's work. The committee appear to take an active interest in the association, and the meeting was unanimous in the election of the Marchioness of Lothian to the office of president.

### NURSE BONE-SETTERS.

What next? A correspondent sends us the following advertisement, which appeared in a local paper in the north of England a few days ago: "Nurse M'Turk, bone-setter and chiropodist, Broughton Street (first street past Derby Hotel), off Padiham Road, Gannow Top, Burnley. Dislocations painlessly reduced. All kinds of skin diseases, bad legs, &c., skilfully treated. All females should consult her. Twenty-two years' hospital training." It would be interesting to hear at what hospital this "nurse" received her "twenty-two years' training."

### THE LITTLEBOROUGH NURSE.

The Littleborough District Nurse Association has issued its third annual report, which contains a summary of valuable work, 206 patients having been visited in their own homes by Sister Lucie during the year. The cases consist of many acute illnesses as well as accidents, and, as the report says, "only those who see the sick poor in their own homes can know how changed is the aspect of the sick room when the nurse has paid a few visits, and how cheered ths patient is by her skilful and kindly ministrations." Increased subscriptions and gifts of old linen are urgently needed to enable the energetic committee of this association to carry out the work in which they are heartily interested.

### VERY HELPLESS.

The patients at Newtownards Union Infirmary include cases of pneumonia, fractures, consumption, epilepsy, "the usual hospital and infirmary patients, and the helpless paralytics," reports the Local Government Board for Ireland. Some 125 of these heavy cases are, says the local press, under the charge of one nurse and one probationer, another probationer being responsible for the whole of the night work. The only help attainable is that of paupers, "often of a low class, both as regards efficiency and morality." The Board recommended the Guardians to consider the advice of the medical officer with respect to the increase of the nursing staff, but most of the Guardians wish the matter postponed until some decision on structural alterations has been arrived at. The supply of adequate nursing may, indeed, be put off, but acute illnesses are not as easily postponed, and the paupers at Newtownards Union Infirmary are indeed to be commiserated.

### SISTERS AT MALTA.

On October 14th the formal opening of the "Little Company of Mary" took place at Malta. The convent has been established to supply Roman Catholic nursing sisters to residents on the island. The ceremony was performed by Archbishop Pace, assisted by Mgr. Debous and others.

### CIVIL HOSPITAL, ST. HELENA.

Miss Rose Blennerhassett will probably leave St. Helena in January, and she may be succeeded by someone from Kimberley, as the passage money thence is much less, and nurses trained there understand the sort of life better than English nurses. The matron at St. Helena dispenses and compounds for the hospital and the out-patients, as well as for the Government officials. This is one more evidence that a knowledge of dispensing is often most essential to a nurse who aspires to promotion, especially if she wishes to try the Colonies.

### POONA.

The report of the Mofussil Nurses' Association was presented at the annual meeting held on September 25th, at Gymkhana. The Honourable Mr. Crowe presided, and the Honourable Mr. Lee Warner, in moving the adoption of the report, remarked that the association had been launched three years ago under the patronage of its warm supporter Lady Harris; he also asserted that "their original object had been steadily kept in view, namely the provision, as far as possible, of nurses for Europeans suddenly taken ill at up-country stations, who, on application elsewhere, could not secure the help they needed."

### KIMBERLEY HOSPITAL NURSING.

An esteemed correspondent, who has had a greater experience of Cape Colony than probably anyone else, and who has an intimate knowledge of all the nursing institutions and hospitals within it, assures us that Dr. Draper Bishop has been too hard upon Sister Henrietta, and that his statements are unfair to her. Kimberley Hospital is Sister Henrietta's creation, and her work in many respects has been admirable. Johannesburg has heaps of money and a large hospital nursed by Roman Catholic nuns; Cape Town, Durban, and Port Elizabeth are all wealthy, but none have been able to create an institution like Kimberley. It follows that Sister Henrietta is entitled to all honour for her work, and deserves great credit besides. We have refrained from commenting on the correspondence until we knew all the facts, and it affords us pleasure to be able to bear this testimony in favour of Sister Henrietta.

### SHORT ITEMS.

At Mullingar Asylum, Ireland, the Governors have agreed to appoint a woman doctor as medical assistant for the female patients.—A hospital with twenty beds has been opened in Edinburgh for the training of Scotch deaconesses and nurses.—A recent American sale at Sutton, near Birmingham, resulted in the sum of £80 being divided between the District Association and the House of Rest.—Only one application is said to have been received for the head nurse's post at the Bromley Workhouse, and her age and qualification were inadequate.—A committee has been appointed to consider the question of a trained district nurse for Penryn.—The sum of £200 was raised towards the funds of the Leaf Homœopathic Hospital by a bazaar held at Eastbourne last week. One of the stalls was presided over by the nurses, and was entirely furnished by contributions from grateful patients.—The Stonehouse District Nursing Association, affiliated with the Queen's Jubilee Institute, has issued a satisfactory balance sheet, and the committee speak highly of the work of Nurse Taite, whose services are universally appreciated.—Mrs. Hilton has published the twenty-third report of her crèche, and appeals for more subscribers.—The certificates and medallions were presented to the Charlton classes of the St. John Ambulance Association by Lord Sandhurst, the Director and Chairman.—The Archbishop of Canterbury, the Earl of Ancaster (Chairman of Executive Committee), and Mr. W. H. Collingridge (the vice-chairman) have been made additional trustees of the National Society for the Prevention of Cruelty to Children.—At the annual meeting of the St. Alban's Diocesan Nursing Institution it was decided that steps should be taken with regard to the provision of more suitable quarters for the Home.—Miss Beatty has been elected Fever Hospital Nurse at Cork.

### Notes for Nurses on Antiseptic Surgery.

By William Horrocks, M.B., F.R.C.S., Hon. Surgeon to Bradford Infirmary.

### IV.—PREPARATION FOR OPERATION.

Having discussed the principles and early methods of antiseptic treatment we proceed to describe briefly the usual procedure at the present time in operation cases.

*Preparation of the Skin of the Patient.*—The skin around the field of operation is well scrubbed with soap and hot water, and hair removed by shaving the part. It is advisable first to wash the part with benzine or turpentine, to remove all grease. For at least an hour before the operation the skin is covered by a towel wrung out of 1 in 20 carbolic lotion.

*Preparation of Instruments.*—After each operation the instruments are washed in soap and water, then sterilised by boiling in a solution of bicarbonate of soda, a drachm to the pint. They are then dried on a clean towel and kept in a well-closed cupboard until required again. Before they are used they should be once more boiled, then placed in sterilised water or carbolic lotion ready for use. The instruments should be made of metal (as the wooden handles are loosened by boiling) and free from nicks. Knife blades should be dipped for a few minutes in the boiling sterilised fluid and are then ready for use.

*Hands of the Operator, Assistants, and Nurses.*—The hands should be carefully scrubbed with soap and water, then immersed for two minutes in 1 in 40 carbolic acid lotion. Special care should be given to the nails and skin about the bed of the nail. The hands should be dipped in lotion from time to time during the operation.

*Sponges.*—Sponges from their structure are always a source of danger. They require the greatest care in their preparation; when possible some substitute should be used. Round Turkey sponges, with small even-sized holes, or flat sponges without detached parts, should be selected. These should be well washed in clear water until the bulk of the sand is removed. They are then placed for three or four days in water, acidulated with hydrochloric acid, until it tastes acid to the tongue. The water is changed once or twice daily. When they are soft and free from grittiness the acid is washed out in clear water. A solution of hyposulphite of soda (half-pound to the gallon of water) is made, and 10 to 12 sponges placed in it. Eight ounces of oxalic acid crystals are added. Sulphurous acid is liberated, and sulphur deposited in the meshes of the sponges. The bleached and disinfected sponges are now washed free from sulphur and stored ready for use in weak carbolic lotion. Sponges which have been used in a foul, septic case are a source of special danger, and should be destroyed after the operation. As substitutes for sponges various things have been used. Mops are made by enclosing pledgets of cotton wool in gauze covers. These should be steeped in 1 in 1,000 corrosive sublimate lotion for twenty-four hours before the operation. They are wrung dry out of the lotion, and passed to the surgeon on a clean dish, not held in the nurse's hand. During the operation sponges or mops are washed roughly free from blood, then steeped for a few minutes in 1 in 1,000 corrosive sublimate lotion, then wrung dry, when they are again ready for use during the same operation. Flat pieces of gamgee tissue, with the edges stretched over, may be used. They are prepared like the mops.

### The Operation Theatre and its Adjuncts.

The operation-room should be free from dust and smells, it should be well-ventilated during the operation. The patient is anæsthetised in an adjoining room, and wheeled or carried into the operation-room. The part to be operated on is now exposed by removing the antiseptic towel by which it is covered. Towels dipped in 1 in 1,000 corrosive sublimate lotion are arranged around the field of operation to prevent the hands of the operater or the instruments touching uncleansed skin or lying on fluffy blankets. The towels should lie on pieces of thin macintosh to prevent them from wetting the patient. The skin of the patient is now sponged over with carbolic or mercurial lotion and the incision made. During the operation the wound is sponged as little as possible, the sponges being dipped in very weak lotion (1 in 6,000 corrosive sublimate, or 1 in 100 carbolic lotion) or in sterilised water. During the operation the vessels are clamped with Spencer Wells' forceps, which are left on for a short time. The larger vessels are tied with catgut ligatures. After all bleeding has been stopped, the margins of the wound are brought together with silk-worm gut sutures. Skin about the wound is dried with a clean, dry sponge, care being taken not to infect the wound by touching the uncleansed skin. The wound is powdered thickly over with iodoform, or a mixture of iodoform and boric acid. Next the wound is placed a layer of sterilised or corrosive sublimate gauze wrung out of weak corrosive sublimate lotion. Outside the gauze a thick packing of wool is placed, that nearest the wound being sterilised or corrosive sublimate wool, that outside ordinary absorbent cotton wool. The dressing is fixed in position by soft muslin bandages.

*Applications, gut, and dressings.*—Silk-worm gut (fishing gut) is prepared by killing the silk worms, just before they begin to spin, in strong acetic acid. The silk is drawn out in long threads from the silk gland, and allowed to dry while stretched betwen two pins on a board. It is a non-absorbent suture, which undergoes no change in a wound. It should be kept ready for use in a vessel containing 1 in 1,000 corrosive sublimate lotion.

*Iodoform* is a yellow crystalline or amorphous powder. It should be sterilized by boiling in water, and then dried, to make its use perfectly safe. It may be used alone or mixed with equal parts of boric acid.

*Sterilised gauze or wool* is prepared by submitting thin layers of the gauze or wool for some time in a closed chamber to superheated steam. The steam is then turned off, and the articles dried by heated air. The gauze and wool are stored in boxes with well-fitting lids.

*Corrosive sublimate lotion* is prepared by dissolving one part of mercuric chloride, or corrosive sublimate, in 1,000 or 2,000 parts of water. The solution is colourless, but may be coloured for purposes of distinction with litmus or aniline blue. Corrosive sublimate gauze or wool is prepared by soaking gauze or wool in this lotion.

### The Poor of Spitalfields.

A trained nurse from the East London Nursing Society works amongst the sick poor in Spitalfields, and during the last year she paid 3,336 visits to 502 patients. "The large proportion of recoveries is encouraging" says the annual report. The majority of these cases are women who dare not avail themselves of the advantages offered in a hospital because they could only secure their own comfort by "breaking up the home." So, whenever possible, medical attendance and skilled nursing in their own homes is eagerly sought by the poor dwellers in Spitalfields. Hospital letters and tickets for convalescent homes are gladly received by the Rector of Spitalfields, and he needs help in money, voluntary workers, and gifts of all kinds of clothing, books, &c., for the inhabitants of this densely crowded district.

### Nursing in Germany.

(Communicated.)

A Convalescent Home destined for consumptive patients of limited means, artisans, workmen, subordinate officials, &c., has been opened at Gröbersdorf by the generosity and untiring energy of Dr. Weicker. The air in this mountainous region is wonderfully pure and exhilarating. The inclusive terms are M.25 weekly in summer, and M.27 in winter. Special care will be taken that only persons in the early stage of consumption are admitted into the institution, and a stay of six weeks will be enforced in order that the patient may derive real benefit from the change and treatment. One day a week is set apart for the thorough examination of each patient, but anyone in need of advice, though not an inmate, may take advantage of the consulting hours.

Special railway tickets are already issued on all the Prussian lines to persons visiting the home, and next year they will be available on all German lines.

Much has already been done towards the organisation of the Deaconesses' Seminary which is being built, adjoining the General Hospital, at Elberfeld. The arrangement is for the pupils of the seminary to be lodged and boarded in the institution, to receive instruction in medicine and nursing in the hospital to which they will give their services. They must submit to its regulations, and fulfil their share of the daily duties of the wards, with the exception of rough work. Their uniform will consist of dark washing dresses, caps, and aprons; the former they find themselves, but caps and aprons are supplied to them. All the pupils will be under the control of a committee, and under the direct supervision of a lady superintendent or matron. The committee is composed of medical officers, and ladies and gentlemen belonging to Elberfeld and the neighbourhood, whose duty it will be not only to control and arrange, but also to take an interest in each pupil, and this is not to terminate with the year of probation.

All evangelical women above twenty years of age and under forty are eligible, provided they are found suited to the work. They must understand housekeeping, the care of children, and bear irreproachable characters, sound health, and have received a good education. They will be instructed by the medical staff in theoretic and practical nursing and hygiene.

Before appointment they will be subjected to four or six weeks' probation, and if proved satisfactory they will then enter on their year's training. Should a pupil voluntarily leave the seminary before the expiration of the year she must pay at the rate of M.50 per month towards her board. Holidays will be arranged, and time for daily exercise set apart and enforced. Each pupil has half Sunday free, regular attendance once a day at church on Sundays and holy days is expected, though not enforced.

The year of the probationer's training is followed by an examination, after which, should the ex-pupils or deaconesses desire it, they can be enrolled as members of the society with the consent of the committee. Each deaconess will be paid a salary sufficient to keep herself and to help towards supporting her relatives if necessary. All who belong to the society are to wear a badge (a brooch is proposed), which on withdrawal from the connection must be relinquished.

The most important matter to ensure the success of the society is to provide it with the necessary funds, not only to start and support it year by year, but to provide pensions or annuities for the deaconesses when old age and sickness creep on. All interested in the undertaking are already seeking yearly subscribers. An annual donation of at least M.1 must be paid by all who belong to the society. No nurse is to be called from a post by the committee without her consent, neither need she go to any hospital or case except by her own wish.

It is proposed to provide a Home of Rest for the deaconesses.

Professor von Helmholtz, the great physician and scientist, passed quietly away on September 8th at his villa at Charlottenburg. The distinction "von" was granted him by the present Emperor on the fiftieth anniversary of his doctorate.

A Munich lady signing herself "Laura Medicus," describes a bed garment which she has herself designed for the use of the sick, and wounded, and which, if introduced, will probably prove of real service. It resembles an ordinary shirt, except that it is made to open all down the front, one side of the sleeves, and the front of the yoke. Thus, the shirt may be opened out, the patient placed on it, and the open parts tied. Tape strings are preferable to buttons as being more comfortable. In cases of serious illness or wounds it will afford relief to the invalid to be spared the necessity of raising the arms, &c., and will be convenient for the doctor who examines him.

The Sanatorium in Gütergotz which has been erected and furnished at the cost of the Berlin Invalid and Old Age Insurance Society, has been opened, and offers to eligible members board, lodging, and nursing free. Visitors are allowed to remain for several months if their health requires it. The need for such a place has long been felt, and some time ago Herr Geheimrath v. Tiemssen wrote an article on the subject in the *Medicinische Zeitschrift für Krankenpflege.* The Sanatorium is situated in a bracing and healthy locality, in the midst of a lovely park. The medical arrangements being left to Professor Kenvers will undoubtedly be of a high order. Herr Director Merke superintends the domestic arrangements, which give proofs of able administration.

### The Royal British Nurses' Association.

The quarterly council meeting of the Royal British Nurses' Association was held on October 15th, and the report of the Executive Committee was received. The chief business transacted by the council meeting was the election of new members on the Executive Committee—Dr. Octavius Sturge, Mr. Page, Dr. Howard Barrett, Miss Eliza Cartwright, and Miss Hughes (Kensington Infirmary)—who will take the places of the following members, who retired by rotation: Sir William Priestly, Mr. Langton, Mr. Alfred Cooper, Dr. Gage Brown, Dr. Galton, Miss Ridley, and Miss De Pledge. The treasurer and the two honorary secretaries were re-elected.

### Presentations.

Dr. F. J. H. Coutts received a handsome brass telescopic lamp from the nursing staff on his departure from the Victoria Hospital, Burnley, and was presented at the same time with a gold-mounted pen by the female patients.

On leaving the Birmingham City Asylum, Miss Frances Parkes, the head nurse, was the recipient of many cordial congratulations on her approaching marriage, and about fifty presents from the numerous friends whose affection and esteem she has enjoyed during the ten years she has worked in the institution.

### Appointments.

Westheath Infectious Hospital.—Miss Mary Elizabeth Hewlett, who was trained at the General Hospital, Birmingham, has been appointed Matron of this hospital, and we wish her every success in her new work.

### Everybody's Opinion.

[Correspondence on all subjects is invited, but we cannot in any way be responsible for the opinions expressed by our correspondents. No communications can be entertained if the name and address of the correspondent is not given, or unless one side of the paper only be written on.]

### HEALTH RESORTS.

"Nurse E. H." writes: Can any reader of The Hospital give me information as to Leghorn? Is it a suitable health resort for consumptives, and is living very costly there? I shall be glad to hear anything about the place, and also whether there is likely to be any opening for private nurses there?

### OVER THE HOSPITAL WALL.

Miss M. Fletcher, Matron, St. William's Hospital, Chatham, writes to us as follows: In justice to those who read my note of warning in The Hospital of October 20th against one calling herself Catherine Bassett, also to prove how untrue and unworthy of credence are her assertions, I would say in the first place the committee were not aware she was applying under an assumed name. Her application was signed Catherine Bassett, so also her wage receipts. As to the letter she accuses me of reading, I saw the letter partly written lying on the house kitchen floor—it must have dropped from a copy of The Hospital Nurse Bassett had lent me—and was not seen by me until quite a half hour after I had been in the house. I knew not whose it was, but at a glance I saw someone who had an invalid husband was applying for a post as nurse. It was Nurse Bassett herself who told me, after she thought I had seen the letter, th t she had been married, but that her husband had died insane shortly after marriage; and to one of the probationers she said, "What a fool I have been, the matron will see I am a married woman." Again, she says she wished to be taken before the committee; this is untrue. Our committee met at the hospital on September 29th, and as usual went through the wards, including the one over which Nurse Bassett had charge. Being asked by one member of the committee why she was staying so short a time, her answer was, "I am very comfortable, but the young man I am engaged to has been employed on the water, but now having a situation on land we shall be married shortly." This was said four days before the finding of the letter. She does not deny getting her box away by stealth, helped by the porter since discharged, but forgets to tell the public her box contained the infected clothing worn by her in the wards, which she has since been compelled to have disinfected. The curator was in the house and grounds from five in the afternoon, and would have known the box was being removed had not Nurse Bassett bribed the porter to convey, with her assistance, her box past the back of the wards, and over the hospital wall. I may say it was owing to the timidity of the nurses that the ordinary policeman on duty in the district was asked to look round the place as we are open and exposed, not to watch the nurses, although it may be seen how much some need it.

### NURSES' HOLIDAYS.

"One Who Wishes to Help Others" writes: You say you would like to hear of cheap trips for nurses, and perhaps this little tour may be new to someone. A friend and I started from Southampton for Havre one April day and slept at Rouen, visiting all the old churches, proceeding next morning to Chartres. This was a tedious whole day's journey, but well repaid the fatigue. The ancient and unique cathedral is even superior in interest to that of Cologne, the houses are very quaint, the walks lovely, and many fine old chateaux are quite near. We found the Hotel du Grand Monarque cheap and good, and from thence returned to Caen, a most fascinating old city. The hospital there should certainly be visited, and of course no one would omit the churches. What admirable taste William and Mathilda appear to have had. We next moved on to Bayeux, where the Sisters of the Couvent du St. Sacrament treated us very well for the modest sum of £1 1s. a week each, and we returned home by Cherbourg, the whole fortnight having cost about £7. A most delightful little trip can be made from Dover to Ostend, third class only 27s., and from Ostend to Bruges, 1s. 2d. The "pensions" are very fair at five francs a week, and excursions can be made to Ghent, Yprès, Huyst, and many interesting places, the fares are so low on the Belgian lines. Indeed, the Hospital St. Jean, the Memling Pictures, and the bridges alone would repay the journey without going beyond Bruges. I will gladly give any other information in my power should it be required.

### Nursing in the U.S.A.

### THE NEW YORK CITY TRAINING SCHOOL.

The report of the New York City Training School for Nurses, at Blackwell's Island, records sixty-six pupils now in course of training, four supervising nurses, a superintendent, and an assistant superintendent completing the staff. They undertake the nursing of the City, Maternity, Gouverneur, and Harlem hospitals, and the superintendent is therefore responsible not only for training nurses in the varied branches of work, but for the efficient nursing of four distinct hospitals.

The training school, under the able management of Miss Louise Darche, accomplishes the twofold object in her own words, "by grading the work in the hospitals into that which is most responsible, responsible, less responsible, and least responsible; and by placing nurses to fill these graded posts of responsibility, according to their experience and ability, all managed under a system of constant surveillance and practical instruction, and so arranged that the nurse has already acquired a knowledge of the next step higher before she is allowed to take it."

The nursing home is situated in its own grounds, and has forty-five bed-rooms, four bath-rooms, two dining-rooms, two store-rooms, cloak-room, trunk-room, large library, or parlour, class-room, study, and a private sitting-room, and the usual offices.

The Training School supervises the housekeeping as well as the nursing of the four hospitals, and has therefore authority over all the "female helps" employed. Miss Darche and Miss Kimber, her assistant superintendent, visited Europe last summer, leaving the school in the hands of Miss Hallinan, who ably fulfilled the duties for three months. Of the tour Miss Darche writes: "One of the most pleasant features of our trip was the privilege of an interview with Miss Nightingale, who expressed the warmest interest in our work as American nurses, and in our own individual school."

Since March 1st of the present year the responsibility of nursing the male patients of the City Hospital has been in the hands of Miss Darche, and the training of twenty-two young men is now under the immediate supervision of a graduate nurse, instead of a head-orderly.

The course at the New York City Training School lasts for two years; candidates are received between the ages of twenty-one, and thirty-five. The entrance examination tests the applicants in reading aloud, in writing legibly and accurately, in simple accounts, and in taking notes of lectures. During the month of trial the superintendent decides as to their practical fitness for appointment as pupil nurses. The nurses are on duty from 7.30 a.m. to 7.30 p.m., an hour being allowed for dinner, and such additional time for rest and study as the work permits. They have half a day off duty once a-week, and, generally, every second Sunday, with a fortnight's annual leave.

There is a registry for "graduates of the school, in good standing," which is under a committee, and provides the public with private nurses at fees of 21 dols. to 25 dols. per week, according to the case.

### The Duchess of Albany.

H.R.H. the Duchess of Albany, whose interest in nursing matters is well known, has graciously consented to open the New Home of the Kingston Nursing Association in Birken head Avenue, Kingston, on December 3rd.

### Asylum News and Nursing.

[Contributions to this section should be addressed to THE EDITOR, 428, Strand, W.C., and have the words "Asylum News" written in left-hand bottom corner of the envelope.]

### AN ASYLUM FROM WITHIN.

LETTERS FROM AN ASYLUM NURSE.—1. FIRST IMPRESSIONS.

I felt very nervous when my cab stopped in front of a large, well-lighted entrance-hall, but it was too late to retreat. In a few minutes I was being interviewed by the matron of the establishment. After kind inquiries as to my journey, she ordered my keys to be given to me, and sent for a nurse to take me to my room. A pleasant-looking girl in a neat uniform of black and white appeared, and conducted me along what seemed to me to be endless passages. Presently we entered a large room, which my conductress said was one of the wards. To my relief, and at the same time disappointment, it was unoccupied and nearly dark. My room opened off a corridor leading from this ward. It was very tiny, but neat and beautifully clean. A bright fire threw out a cheerful glow, and my little trunk seemed quite like an old friend.

My conductress who had been chatting in the most friendly manner now left me, saying that something to eat would be sent up immediately. Very soon a tray containing tea, bread and butter with ham and egg appeared, and being very hungry, I did full justice to the meal. My next step was to make a thorough survey of my little room. I looked in, and under the bed. I even peeped into the chest of drawers, and, being satisfied that no lunatic was concealed anywhere, locked the door and sat down in front of the fire. I was feeling very lonely, tired and nervous; my imagination magnified every sound to a most alarming extent. To speak candidly I was in what my young brother would call "a beastly funk," and was fully convinced that I had made a great mistake in coming here. To get out of these feelings I jumped up, unpacked my box, put out all my little treasures, slowly undressed, and got into bed. Here I indulged in a good cry which must have done me good, for I fell asleep.

How long I slept I cannot say, but I was awakened by the most fearful screams, which seemed to be in my very room. I started up, trembling in every limb, and felt as though I were gasping for breath. Scarcely conscious of where I was I got out of bed, and stood shivering and listening by my bedside, but afraid to move. I heard steps hurrying past—presently came a murmur as of some one talking persuasively, and gradually the screams ceased, though a muttering sound was kept up for a long time afterwards. Some one besides myself seemed to have been disturbed by the noise, for this muttering was punctuated by an occasional volley of bad language from some one on the other side of me.

I was now wide awake but thoroughly chilled and frightened. Getting into bed again I made a vow to pack my box and start off home by the first train in the morning. Considerably cheered by this resolution, in time I fell asleep and was awakened in the morning by a knocking at my door. I jumped up in a fright calling out "Who's there?" when the door was unlocked by my conductress of the previous evening. She asked me how I had slept, and cut short my dreadful story of the night's horrors by smilingly remarking "Yes, it must have been trying for a stranger. Poor old Maggie had a bad night." She had brought me my breakfast and told me that a new nurse was not expected on duty until after breakfast on her first morning. I dressed hastily and before eating my breakfast took a hurried look outside my door. To my astonishment I found that I was gazing into a bright cheerful room, decorated with plants and flowers; the walls covered with pictures and ornaments, and the room inhabited by a number of neatly dressed, and to all outward appearance, sane women.

### NORTHAMPTON COUNTY ASYLUM.

The following nurses have passed the examination at the end of their third year's training, and have received certificates: M. Spencer (silver medallist), H. Sensicle, E. Rylett, M. Bateman, and C. Edwards. The committee of this asylum now give £1 a-year additional to every nurse holding the certificate.

### GRAHAMSTOWN ASYLUM, CAPE COLONY.

It is not often that journals printed and published in asylums can be called lively reading. We think the staff at Fort England, Grahamstown, are to be congratulated upon the *Fort England Mirror*. This little magazine speaks volumes for the energy and enthusiasm of Dr. Greenlees, the medical superintendent, and here, at least, the Old Country may take lessons from her children beyond the sea. There is always a danger of such papers sinking into personalities or absolute dulness, but hitherto the Grahamstown editor has steered his course safely between these rocks.

### DERBY COUNTY ASYLUM, MICKLEOVER.

We regret to hear of the death of Mr. H. Bird, chief attendant of this asylum. While assisting in the postmortem room he contracted blood-poisoning, and succumbed after an illness of less than a week. He is greatly regretted by staff and patients alike. Workers in asylums are peculiarly liable to accidents, and often suffer severely under the mental strain caused by their anxious duties. Mr. Bird was singularly unfortunate, in that, in addition to running the risk of these dangers, he fell a victim to one which, we would suppose, was more likely to attack the medical officers. It is sad that no legal provision has been made to enable the authorities to grant gratuities to those dependent upon an officer, who has been thus virtually killed in the execution of his duty.

### Male Nurses' Temperance Co-operation.

THIS establishment in Great Marylebone Street undertakes to supply attendants who have had experience with sick people, and temperance is strictly guaranteed in the men sent out to cases. It is unfortunate that no training in general nursing can be obtained in England by male attendants, who are therefore seldom able to attain to the position of the qualified nurse.

### Notes and Queries.

### Queries.

(26) *Male Attendant.*—Can you tell me how to procure the last three chapters of "Mental Nursing," by Dr. Harding? They are not in my copy.—*A. T.*

(27) *Copenhagen.*—Information wanted about hospitals in this city, especially with regard to nursing.—*A. T. R.*

(28) *Preparations.*—Can I have an opinion as to the merits of a certain preparation inserted in the columns of this journal?—*W. A. M.*

(29) *Co-operation.*—Can you tell me the address of the head office of the Nurses Co-operation?—*Marie.*

(30) *Patent.*—Can you kindly give me address of place where a nurse's invention can be patented?—*Inventive Nurse.*

(31) *L.O.S.*—Is there any class held in preparation for the London Obstetrical Society's January examination which a nurse can attend?—*Nurse K.*

### Answers

(26) *Male Attendant* (*A. T.*).—The second edition of "Mental Nursing" contains three new chapters; the book is 2s. 6d.

(27) *Copenhagen* (*A. T. R.*).—We should advise you to write direct to the "Commune Hospital," and to the Royal Fredrik's Hospital, both in Copenhagen.

(28) *Preparations* (*W. A. M.*).—You had better consult a doctor in your own neighbourhood on the subject.

(29) *Co-operation* (*Marie*).—The only address of the Nurses Co-operation is 8, New Cavendish Street, London.

(30) *Patent* (*Inventive Nurse*).—Patent Office, 25, Southampton Buildings, London, W.C.

(31) *L.O.S.* (*Nurse K.*).—Yes, at 12, Buckingham Street, Strand. Write (enclosing stamped envelope) to the Hon. Secretary of the Midwives and Trained Nurses' Club at that address.

For Book World for Women and Nurses, Reading to the Sick, and Minor Appointments, see overleaf.

### The Book World for Women and Nurses.

[We invite Correspondence, Criticism, Enquiries, and Notes on Books likely to interest Women and Nurses. Address, Editor, THE HOSPITAL (Nurses' Book World), 428, Strand, W.C.]

MEDICAL NURSING. Notes of lectures given to the probationers at the London Hospital by the late Dr. James Anderson, M.D., F.R.C.P. Edited by ETHEL F. LAMPORT. (Published by H. K. Lewis, 136, Gower Street, London, price 2s. 6d.)

THIS little volume will form a welcome addition to many a nurse's private library. It is exceedingly well got up, as regards type and paper, and the index, in itself no bad test of thoroughness, is very complete. In the biographical notice with which the book begins Sir Andrew Clark has left his testimony that "Dr. Anderson was a man of exceptional character, ability, and promise. As a teacher he was popular and successful, from his undergraduate days in Aberdeen, when circumstances made teaching a necessity, till later years when it became one of his chief delights." It follows then that the instruction of so enthusiastic a teacher should be interesting and valuable, and the pages of the book now before us are certainly both. In the introduction which was written out by Dr. Anderson himself (and, as the editor tells us, "appears in its original form, whilst the others have required some amplifications"), he expresses a wish that his lecture "will rather pertain to a pleasant conversation as between friends." Probably this custom of treating pupils as intelligent friends partly accounts for the enormous personal influence which the late doctor possessed. His habit of invariably assigning the best motives to his fellow-creatures was calculated to engender in most of them a desire to deserve his good opinion. He accredited all nurses with the steadfastness of purpose so characteristic of himself, and told them, "You are to be earnest, wise, and kindly helpers to your patients, loyal and intelligent co-workers with your physician. . . ." "You can never really do properly what you are bid machine-fashion. You must have a reason, and it will be part of the object of these lectures to help you in this respect, but they can help you only if you give good honest work to it yourselves. And this work must be earnest." Every sentence in this introductory chapter is pregnant with meaning to the last word, "A soldier obeys orders, but he is a better soldier if he knows why the orders are given; and it is exactly the same with a nurse." Not only the subjects of food and digestion, but the administration of the former are duly considered, and the hints are of universal application; many a private patient would fare better if his friends had studied the refinements of service which are here given under the head of "Feeding the Patient." In the chapter devoted to the respiratory system attention is called to the kind of ventilation desirable for patients with lung disease, and each lecture has some special points of practical interest to commend it to probationers eager for easily-digested knowledge. Many friends will feel indebted to Miss Lamport for the care and patience which she has devoted to developing the abbreviated notes into so worthy a memorial of the late Dr. James Anderson.

DISINFECTION BY OXYGEN.

A little pamphlet bearing this title contains a report on the germicidal and disinfecting action of Condy's fluid. It is by A. B. Griffiths, Ph.D., F.R.S. (Edin.), F.C.S., and gives the particulars of nine series of experiments made by the author with this fluid. It is published by H. Grevel and Co., London.

THE ZENANA, OR WOMEN'S WORK IN INDIA.

The copy of "The Zenana," which we have received from the hon. secretary, Vol. I. for 1894, is a prettily got-up book containing a record of the work done by the Bible and Medical Mission in India. It is published by Messrs. S. W. Partridge and Co., 9, Paternoster Row, price 2s.

### For Reading to the Sick.

SUNDAY.

**Motto.**

The pearl of days.—*Anon.*

**Verses.**

The Sundays of man's life,
Threaded together on Time's string
Make bracelets to adorn the wife
Of the eternal glorious King.—*George Herbert.*

How many blessed groups this hour are bending,
Through England's primrose meadow paths, their way
Towards spire and tower midst shadowy elms ascending,
Whence the sweet chimes proclaim the hallowed day!
The halls from old heroic ages grey
Pour their fair children forth; and hamlets low,
With whose thick orchard blooms the soft winds play,
Send out their inmates in a happy flow,
Like a freed vernal stream. I may not tread
With them those pathways—to the feverish bed
Of sickness bound; yet, O my God! I bless
Thy mercy that with Sabbath peace hath filled
My chastened heart, and all its throbbings stilled
To one deep calm of lowliest thankfulness.
—*Felicia Hemans.*

**Reading.**

A subject of trial to many sick people is the Sunday. Some feel this more acutely than others. The want of public worship is, and ought to be, a great trial to you who have been in the habit of assembling together in a place "where prayer is wont to be made." There is a wonderful help in the sympathy of many worshipping together; our sluggish souls need every help. And when prayer and praise are offered the heart is lifted up with others, and at least the "voice of melody" ascends. All these blessings are withheld from the sick, and the cheerful sound of the church bells does but remind them that they cannot go up to the house of the Lord. But try and make your Sunday as much like the time when you could go to church. If you know when the service is beginning, then begin to read also, then you may join with all who are worshipping everywhere, and you will cease to feel cut off and isolated. When you have read as much as you can you had surely better lie still for a time and do nothing. Then gladly take the refreshment of seeing any of your family or friends who come to see you. In the afternoon or evening you may be able to read again, and thus you will not find Sunday a tedious or a lonesome day, but will especially enjoy the rest and refreshing it offers, the entire relaxation from the work of other days. The more a love for this day is cultivated it will become a glad day—a day to be reckoned upon all the week, and rejoiced on when it comes. Thus it will become the pledge and foretaste of the rest which they long for, the "rest which remaineth for the children of God." —*Sickness, its Trials and Blessings.*

### Minor Appointments.

VICTORIA HOSPITAL, BURNLEY.—Miss A. Maud Jones has been made night superintendent of this hospital, in which she received three years' training, and afterwards held the post of sister in the children's and female wards and theatre.

